# Influence of inflammatory arthritis on leukocyte esterase strip results in the diagnosis of periprosthetic joint infection

**DOI:** 10.1186/s13018-019-1523-0

**Published:** 2020-01-10

**Authors:** Jinling Zhang, Binjie Gui, Fangyue Cheng, Genxiang Rong, Zhi Tang, Cailiang Shen

**Affiliations:** 1 Department of Orthopedics, The First Affiliated Hospital of Anhui Medical University, 218 Jixi Road, Hefei, Anhui 230022 People’s Republic of China; 2 Departments of Rheumatology, The First Affiliated Hospital of Anhui Medical University, Hefei, People’s Republic of China

## Abstract

**Background:**

The leukocyte esterase (LE) strip is considered as a helpful method to detect infection, which might be influenced by other inflammatory diseases. This study aims to explore whether the centrifugation of synovial fluid could influence the positive result of LE strip caused by inflammatory arthritis during the diagnosis of periprosthetic joint infection (PJI).

**Methods:**

From March 2016 to December 2018, 64 patients who were diagnosed as PJI or aseptic arthritis and another 20 patients with inflammatory arthritis were enrolled in our study. After synovial fluid samples were obtained, the LE strip test was performed with and without centrifugation. Then clinicians read the color changes 3 min after the samples were dropped and classify the results based on the instruction of strip. The differences between septic and aseptic arthritis patients and septic and inflammatory arthritis patients were analyzed.

**Results:**

Among the included 21 PJI samples, 19 of them showed positive results (++) of LE strip before centrifugation. After centrifugation, two samples changed from two-positive (++) to one-positive (+), which is also considered as positive. Before centrifugation, 29 of the LE strip tests in the aseptic arthritis group (43 samples included) were ++ or +. After centrifugation, 16 of the samples yielded negative results. Among 20 samples with inflammatory arthritis, LE strip of 18 samples were positive (++ or +) before centrifugation, among which only 3 samples remained as positive after centrifugation.

**Conclusion:**

LE strip test results could be influenced by inflammatory arthritis during the diagnosis of PJI. Centrifugation should be performed for LE strip tests to determine whether the result is a true positive or a false positive influenced by inflammatory arthritis.

## Introduction

Periprosthetic joint infection (PJI) is one of the most severe complications of total joint arthroplasty (TJA). PJI biomarkers including elevated erythrocyte sedimentation rate (ESR), C-reactive protein (CRP), and leukocyte esterase (LE) strip test have been reported in recent years. However, among these PJI patients, some are also accompanied with inflammatory arthritis, such as rheumatoid arthritis (RA), psoriatic arthritis (PsA), reactive arthritis, and ankylosing spondylitis (AS) [1]. PJI and inflammatory arthritis sometimes cannot be differentiated simply through symptoms. Timely and accurate differentiation of PJI from inflammatory arthritis is essential since the treatments are quite different. Second-stage arthroplasty is required for treatment of PJI, while steroids and/or immunosuppressive drugs, which could aggravate septic cases, are required for inflammatory arthritis.

The abovementioned biomarkers for PJI are also considered to be available for the evaluation of patients with RA or AS. But there is no consensus about the diagnostic criteria for these patients, which is a challenge for orthopedic surgeons. Synovial fluid culture, white blood cell (WBC) count, and polymorphonuclear percentage (PMN%) can be a key component in the diagnosis of septic arthritis. However, these biomarkers are frequently influenced by the use of antibiotics [2]. Thus, these markers may not be ideal for solving this problem.

Leukocyte esterase (LE) strip test has been used to detect urinary tract infections and was first applied to the diagnosis of PJI by Parvizi et al. in 2011 [3]. Since then, several studies evaluated the diagnostic efficiency of the LE strip [4–6]. However, cases with inflammatory arthritis were always excluded in previous studies, which evaluated the diagnostic efficiency of the LE strip for PJI [7], and thus, the influence of inflammatory arthritis has not been well explored.

When performing LE strip test for PJI diagnosis in our lab, we accidentally found that inflammatory arthritis leads to false-positive LE strip results. In addition, we also observed that these false-positive LE results in patients with inflammatory arthritis could change into negative after centrifugation of synovial fluid. Thus, the abovementioned phenomenon leads to the hypothesis: whether centrifugation could eliminate inflammatory arthritis-induced false-positive LE strip test results in the diagnosis of PJI.

Therefore, the purpose of our current study was to analyze the results of LE strip tests with or without centrifugation in PJI patients and inflammatory arthritis cohorts.

## Methods and materials

### Patient enrollment and grouping

Our study has been approved by the ethics committee of our hospital (PJ2016-06-01). From March 2016 to December 2018, patients who were suspected of PJI from the Department of Orthopedics or patients who were diagnosed with inflammatory arthritis from the Department of Rheumatology and Immunology were included in our study. Patients meeting the following criteria were excluded: not enough synovial fluid samples were obtained, unreadable LE strip test results without centrifugation due to color disturbance caused by blood contamination, and insufficient information for a definitive diagnosis. The synovial fluid samples were collected from these enrolled patients.

PJI was determined based on the Muscularskeletal Infection Society (MSIS) criteria. Patients meeting one of the three following criteria were classified in the PJI group: (1) sinus tract communication with a prosthesis; (2) the same pathogen isolated by culture from two separate fluid samples or tissues; and (3) four of the following six criteria were positive: (1) increased ESR and CRP (ESR > 30 mm/h, CRP > 10 mg/L); (2) increased synovial fluid white blood cells (> 3000/μL); (3) increased synovial fluid percentage of polymorphonuclear neutrophils (> 65%); (4) presence of purulence in synovial fluid; (5) positive culture in synovial fluid or tissue; and (6) histopathological analysis of perisprosthetis showed more than five neutrophils per high-power field on frozen section in more than five high-power fields (× 400).

Patients with any suspicion of inflammatory arthritis were referred to a rheumatologist for further confirmation. Thus, the PJI group and aseptic arthritis group from the Department of Orthopedics and the inflammatory arthritis group from the Department of Rheumatology and Immunology were divided in this current study.

### LE strip test

After aspiration, synovial fluid was immediately dripped onto the LE strip test pads (AUTION Sticks, 10PA, ARKAY, Japan). The LE strip test results were read based on the color change 3 min after the drop. Then the left synovial fluid was centrifuged at 3000 rpm, 5 min before the supernatant was collected and applied to a new LE strip test to obtain another result. The changing color of the test strip was interpreted as negative (white), trace (25/75, slightly purple), one-positive (+/250, intermediate purple), or two-positive (++/500, dark purple). As for PJI patients, we defined intermediate and dark purple as positive results.

The LE results before and after centrifugation were read by three independent observers who had at least 30 cases of experience using LE strip tests. The observers were blinded to any diagnostic information about the patients. If there were any inconsistencies in the recorded results among the three observers, the grade with the highest agreement was considered as the final result.

### Statistical analysis

The statistical analysis was performed using IBM SPSS Statistics (IBM Corp, Armonk, NY, USA), and *P* < 0.05 was considered significantly different.

## Results

Among the cases from the Department of Orthopedics, 43 were defined as aseptic arthritis patients, while 21 were defined as PJI patients based on MSIS criteria. As for the LE strip, a one-positive (+) or two-positive (++) result was considered to indicate a PJI. Before centrifugation, 19 of the 21 PJI cases showed + or ++ results, while after centrifugation, results of two samples change from ++ to +. However, in the aseptic arthritis patients, before centrifugation, LE strip results of 15 cases were ++ and 14 cases were +. After centrifugation, only 1 case maintained as ++ while 2 cases were +, all the remained 26 cases changed to negative results (Tables 1 and 2). Then, we further reviewed the history of these false-positive patients and found that eight of them had been diagnosed as rheumatoid arthritis (RA), three had been diagnosed as reactive arthritis, and two as psoriatic arthritis (PsA). After a follow-up of 3 months, patients of aseptic arthritis group exhibited remission without antibiotic treatment. Thus, this result hinted that inflammatory arthritis might confuse the results of the LE strip, which could be eliminated through centrifugation before performance during the diagnosis of PJI.

**Table 1:** LE strip test results before centrifugation

Diagnosis	Before centrifugation		
	++	+	Negative
PJI (*n* = 21)	19	0	0
Aseptic arthritis (*n* = 43)	15	14	14
Inflammatory arthritis (*n* = 20)	14	4	2

**Table 2:** LE strip test results after centrifugation

Diagnosis	After centrifugation		
	++	+	Negative
PJI (*n* = 21)	17	2	0
Aseptic arthritis (*n* = 43)	1	2	30
Inflammatory arthritis (*n* = 20)	2	1	17

In order to verify this hypothesis, we further enrolled 20 patients who were diagnosed as inflammatory arthritis from the Department of Rheumatology and Immunology. Among these patients, 15 were diagnosed as RA, 3 as AS, and 2 as PsA in combination with AS. The LE strip results showed that before centrifugation, 14 cases were ++, 4 cases were +, and 2 cases were negative. While after centrifugation, 12 ++ cases changed into negative, 2 ++ cases changed into +, 3 + cases changed into negative, and the other one + case remained the same (Tables 1 and 2).

## Discussion

Patients with painful and swollen knee are quite common in orthopedic outpatient clinics. However, this presentation often confuses doctors because these symptoms may be caused by septic arthritis or other intra-articular inflammatory conditions, such as RA and AS. Although serum parameters including ESR and CRP are considered as biomarkers for PJI, they have relatively poor specificity and can be influenced by factors such as extra-articular infection or noninfectious inflammatory disease [8]. In our precious study, we found that the LE strip of inflammatory arthritis could also generate ++ result, which might confuses the diagnosis of PJI. Then, in this study, we further investigated the influence of centrifugation on the LE strip for differentiating PJI from aseptic arthritis such as RA and AS.

Among the many biomarkers in synovial fluid with the potential to diagnose PJI, the LE strip test is unique since it is inexpensive, rapid, and commercially available. However, considering that the LE strip was optimized and developed for urinary testing, it is expected that the test would have altered characteristics in synovial fluid. According to previous meta-analysis, the main problem of LE strip is that the cut-off value is not determined since it is a colorimetric test and many factors including blood contamination can greatly influence the results [9]. Due to the high rate of blood interference, it has been suggested that the sample is centrifuged before dropped on the LE strip. In addition, it has been reported that an invalid test strip is mainly those with high WBC count [5]. In addition, Deirmengian et al. [9] had demonstrated that the urinary LE test strip sometimes fails to detect LE enzymatic activity, even when there is abundant LE in the synovial fluid. They attributed to this phenomenon to the LE inhibitors in inflamed synovial fluid, which may inhibit the LE strip reaction. Thus, a greatly elevated concentration of white blood cells might be required to yield a positive test from synovial fluid. If the sensitivity of the LE strip is 80%, then 1 in 5 patients with PJI might be missed using this test, which generated false-negative results. Thus, more study is required to further investigate the influence factors for the false-negative results of the LE strip.

We hypothesize the results of our current study as the following. Leukocyte esterase (LE) is an enzyme released by neutrophil and it could be found both inside and outside the neutrophils. As for inflammatory arthritis, although neutrophils are recruited to the joint, they might not be fully activated. Thus, only a small amount of LE is released outside the neutrophils around the joint. With centrifugation, these enzymes, which exist inside the neutrophils, might be eliminated from the supernatant. In addition, the synovium may have been partially debrided due to primary total knee arthroplasty in patients who had already undergone a revision arthroplasty. Thus, patients with an autoimmune disease may not present with the typical acute inflammatory change in their knees.

The results of this study indicated the influence of inflammatory arthritis on the diagnosis of PJI using the LE strip test. Joint fluid can provide a variety of clinical information, which has frequently been dismissed by clinicians [10]. This could lead to the inappropriate management of patients with a painful and swollen knee and are accompanied with longer hospital stay [11]. Based on our data, for a painful swollen joint in the outpatient clinic, a LE strip test is recommended to screen for infection along with other synovial fluid tests, such as culture. When the result is negative (negative or trace), septic arthritis could usually be excluded. With a positive result (+ or ++), centrifugation ought to be performed for further evaluation. If it remains to be positive, there is a large possibility that this is a septic arthritis. If the LE strip test is negative after centrifugation, tests related to the diagnosis of inflammatory arthritis and communication with physicians from the Department of Rheumatology are essential.

Limitations of our study include the following: First, the number of samples enrolled in our study is quite limited. A large scale and multi-center study is still required to further investigate the influence of inflammatory arthritis during the diagnosis of PJI with LE strip test. Second, patients may present in various stages of inflammatory disease activity, which could not explain the influence of different disease-modifying immunomodulatory medications. Third, the present study included only knee cases, and whether the same conclusion applies to other hip and shoulder remains to be confirmed.

In conclusion, the LE strip results could be influenced by inflammatory arthritis when used for diagnosing PJI, and this influence could be eliminated through centrifugation before the test. For patients suspected of septic arthritis, the LE strip could be used as a rapid and helpful method for screening. If there is a positive result, additional centrifugation ought to be performed for further determination. Since diagnosis of PJI is always confronted with the interference of autoimmune disease, more synovial fluid biomarkers such as alpha-defensin, CRP, and interleukin-6 ought to be further explored whether they were influenced by inflammatory arthritis and how to distinguish between an acute rheumatological flare and PJI.

## Data Availability

All data generated or analyzed during this study are included in this published article.

## Abbreviations

AS: Ankylosing spondylitis
CRP: C-reactive protein
ESR: Erythrocyte sedimentation rate
LE: Leukocyte esterase
MSIS: Muscularskeletal Infection Society
PJI: Periprosthetic joint infection
PMN: Polymorphonuclear
RA: Rheumatoid arthritis
TJA: Total joint arthroplasty
WBC: White blood cell
